# Polyphenols from *Tamarix nilotica*: LC–ESI-MS^n^ Profiling and In Vivo Antifibrotic Activity

**DOI:** 10.3390/molecules23061411

**Published:** 2018-06-11

**Authors:** Ahmed Sekkien, Noha Swilam, Sherif S. Ebada, Ahmed Esmat, Ahmed H. El-Khatib, Michael W. Linscheid, Abdel Nasser Singab

**Affiliations:** 1 Department of Pharmacognosy, Faculty of Pharmacy, British University in Egypt (BUE), Cairo 11837, Egypt; Ahmed.Sekkien@bue.edu.eg; 2 Department of Pharmacognosy, Faculty of Pharmacy, Ain Shams University, Cairo 11566, Egypt; sherif_elsayed@pharma.asu.edu.eg (S.S.E.); dean@pharma.asu.edu.eg (A.N.S.); 3 Department of Pharmacology and Toxicology, Faculty of Pharmacy, Ain Shams University, Cairo 11566, Egypt; ahmed.esmat@pharma.asu.edu.eg; 4 Department of Pharmaceutical Analytical Chemistry, Faculty of Pharmacy, Ain Shams University, Cairo 11566, Egypt; ahmed.elkhatib@cms.hu-berlin.de; 5 Laboratory of Applied Analytical and Environmental Chemistry, Department of Chemistry, Humboldt-Universität zu Berlin, 10099 Berlin, Germany; m.linscheid@chemie.hu-berlin.de

**Keywords:** *Tamarix nilotica*, Tamaricaceae, polyphenols, HPLC/HRESI/MS/MS, antifibrotic activity

## Abstract

*Tamarix nilotica* (Ehrenb.) Bunge (Tamaricaceae), an indigenous plant to the Middle East region, is well-known as a medicinal plant for treating many human ailments. The current study aimed at exploring the polyphenol profile of the alcohol soluble fraction of aqueous *T. nilotica* extract, assessing its in vivo antifibrotic activity and the possible underlying mechanism, to unravel the impact of quantitative difference of sulphated polyphenols content on the antifibrotic activity of *T. nilotca* grown in two different habitats. Polyphenol profiling of *T. nilotica* extracts was performed using HPLC-HRESI-QTOF-MS-MS. The major polyphenol components included sulphated flavonoids, phenolic acids and free aglycones. The antifibrotic activity was evaluated through carbon tetrachloride-induced liver fibrosis in rats. Biochemical evaluations revealed that both fractions ameliorated the increased levels of hepatic aminotransferases, lipid peroxidation, hydroxyproline, α-smooth muscle actin (*α*-SMA), tumor necrosis factor-*α* (TNF-*α*), cyclooxygenase-2 (COX-2) and nuclear factor kappa B (NF-κB). Moreover, both fractions reduced catalase activity (CAT) and enhanced hepatic glutathione (GSH) content. Histopathological imaging undoubtedly confirmed such results. In conclusion, the *T. nilotica* polyphenol-rich fraction exhibited potential antifibrotic activity in rats. Significant alterations in GSH levels were recorded based on the sulphated polyphenol metabolite content.

## 1. Introduction

Hepatic fibrosis is a consequence of chronic liver diseases such as chronic hepatitis C virus (HCV) infection, alcohol abuse and non-alcoholic steatohepatitis (NASH) [1]. Hepatic fibrosis is characterized by an excessive accumulation of extracellular matrix (ECM) proteins, including collagen as a common wound healing response, distorting the normal hepatocellular architecture and forming a fibrous scar, which subsequently develops into hepatocellular nodules or an irreversible complication known as liver cirrhosis [2]. In developing and developed countries, liver cirrhosis represents an end-stage liver complication where liver transplantation becomes the only treatment choice, which is even not easy to achieve considering the number of donor organs available and the clinical condition of the potential recipients [3]. 

Therefore, research interests have been directed toward hepatic fibrosis several decades ago as the last reversible phase of complicated chronic hepatic diseases before turning into liver cirrhosis [1]. Based on the inconsistent effectiveness of treatment protocols using corticosteroids and interferon in chronic liver diseases and their complications, much attention have been drawn to herbal remedies as a last resort to help regenerating hepatocytes in liver fibrosis [4]. This notion was practically proven by the sudden rise in the popularity and use of herbal drugs by liver patients (up to 65%) within few years due to the fact that herbal remedies represent undoubtedly an easily accessible, affordable and safe alternative to the current treatment protocols which turned out to be explicitly inadequate [4].

The genus *Tamarix* is native to Africa as well as the Eurasian region. The genus comprises over 50 species, including *T. nilotica*, which is an evergreen tree or shrub that can grow up to 5 m [5]. *Tamarix nilotica* has been widely incorporated in the folk medicine of many societies and cultures for the treatment of sores, wounds, spleen oedema or uterus infections, while the extract itself has been used as an antiseptic [6,7].

Former scientific reports proved that *T. nilotica* flowers possess in vivo hepatoprotective properties [8], which inspired this study to compare the antifibrotic activity of the alcohol soluble fraction of an aqueous extract of *T. nilotica* aerial parts from two different habitats, Egypt (ETN) and Saudi Arabia (STN), and their secondary metabolite profiling using HPLC/HRESI/MS/MS.

## 2. Results

### 2.1. Polyphenols Metabolic Profiling

Chemical investigation of the alcohol soluble fraction of aqueous extract of ETN and STN was conducted via HPLC/HRESI/MS/MS and dereplication tools using natural product databases and comparison with the reported literature. Results (Table 1 and Table 2) revealed that both fractions feature the abundance of polyphenol secondary metabolites including flavonoid glycosides, methylated flavonoid aglycones, phenylpropanoids, together with different sulphated compounds.

The negative ion mode profile of ETN showed three major peaks corresponding to methyl ferulate sulphate, (iso)ferulic acid-3-sulphate and coniferyl alcohol sulphate derivative. The [M − H] peak at *m*/*z* 287 and a fragment ion at *m*/*z* 207 corresponding to the removal of a −SO_3_ group were consistent with those reported for methyl ferulate -3-O-sulphate [9]. Isoferulic acid-3-sulphate was identified by its [M − H]^−^ peak at *m*/*z* 273 and a base peak signal at *m*/*z* 193 for the deprotonated phenolic acid [10]. Coniferyl alcohol 4-*O*-sulphate showed [M − H]^−^ peak at *m*/*z* 259 and a fragment ion at *m*/*z* 179 after the loss of its −SO_3_ group [11]. The chromatogram further showed a molecular ion peak at *m*/*z* 299 along with fragment ion at *m*/*z* 284 as a result of the loss of a −CH_3_ group assigned for kaempferide [9]. Gallic acid was identified after showing a peak at *m*/*z* 169 and a fragment at *m*/*z* 125 as described by [12,13]. A molecular ion peak at *m*/*z* 483 with fragment ion at *m*/*z* 313 due to loss of a galloyl moiety proved the existence of nilocitin [14].

Kaempferol 7,4′ dimethyl ether 3-*O*-sulphate was identified by a peak at *m*/*z* 393 and a fragment ion at *m*/*z* 313 indicating desulphonation [15]. A peak of the deprotonated ion at *m*/*z* 315 accompanied by a fragment ion at *m*/*z* 300 due to demethylation were similar to those reported for tamarixetin [9,16,17]. Kaempferol showed a molecular ion peak at *m*/*z* 285 [15,18]. Another peak at *m*/*z* 193 corresponding to deprotonated isoferulic acid showing a base peak at *m*/*z* 178 after the loss of a methyl group was detected [17,19]. Quercetin was identified by a molecular ion peak at *m*/*z* 301 [15,18]. A [M − H]^−^ peak at *m*/*z* 197 representing methyl gallate 4-methyl ether showed fragments at *m*/*z* 182 and 167 indicating successive demethylations [15,18]. Another peak appeared at *m*/*z* 461 corresponding to kaempferol 3-*O*-β-glucronide with a daughter ion fragment at *m*/*z* 285 that belongs to the deprotonated free aglycone [15].

Further examination revealed the presence of 4′-*O*-methyl quercetin 3-*O*-β-hexoside, identified by a [M − H]^−^ peak at *m*/*z* 477 and a base peak at *m*/*z* 315 due to cleavage of the *O*-glycosidic bond release of the free aglycone and loss of the sugar moiety and another fragment at *m*/*z* 300 representing demethylation [16]. Kaempferol 4′-methyl ether 3-*O*-sulphate was characterized by a molecular ion peak at *m*/*z* 379, and the removal of the −SO_3_ moiety was responsible for the base peak at *m*/*z* 299 [11]. Finally, tamarixetin 3-*O*-sulphate was identified by a peak at *m*/*z* 395 and a base peak at *m*/*z* 315 representing the loss of the −SO_3_ group [11,17]. *n*-Feruloyltyramine was only detected through inspection of the positive ion mode showing a major peak of [M + H]^+^ at *m*/*z* 314 and a fragment at *m*/*z* 177 that represents the loss of ferulic aldehyde [20,21]. 

Quantitative differences in the polyphenol profiles of ETN and STN were proved, where kaempferol 4′-methyl ether 3-*O*-sulphate, methyl ferulate-3-*O*-sulphate, isoferulic acid-3-sulphate and tamarixetin 3-sulphate were the major polyphenols of the STN extract. 

### 2.2. Total Phenolic Content

The total phenolic contents of ETN and STN were determined through a calibration curve using gallic acid as a standard. The results (Table 3) are presented in equivalent milligrams of gallic acid per 1.0 g of dried extract and they revealed that STN possesses a relatively higher total phenolic content (111.8 mg GA/g extract) compared to ETN (95.1 mg GA/g extract). 

### 2.3. Oxygen Radical Absorbance Capacity (ORAC Assay)

**Figure 1:**
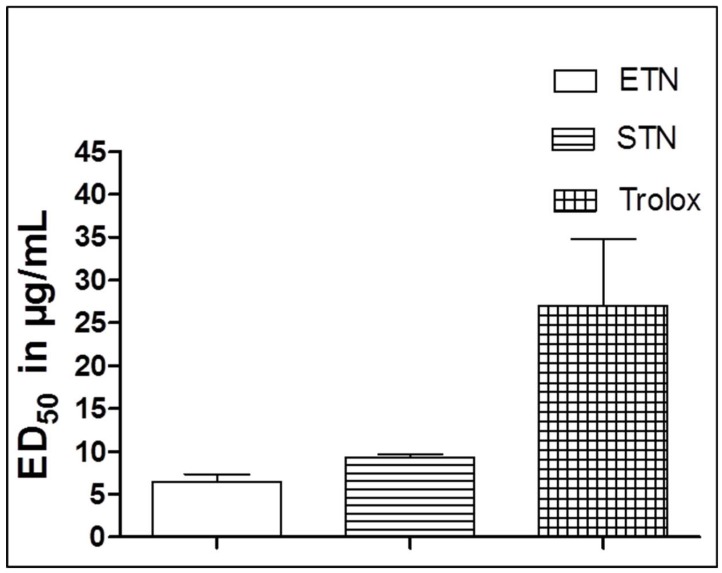
Radical scavenging activity of ETN and STN as compared to Trolox (as a positive control). Results are given as mean values ± SD of *n* = 3.

The antioxidant activities of ETN and STN were tested by the ORAC assay using Trolox as a positive control. Both extracts exhibited a pronounced antioxidant activity with ED_50_ values of 6.38 and 9.32 µg/mL, respectively, which are significantly lower than that of the reference standard Trolox (ED_50_ = 27.0 µg/mL), (Figure 1).

### 2.4. Hepatotoxicity Indices

**Figure 2:**
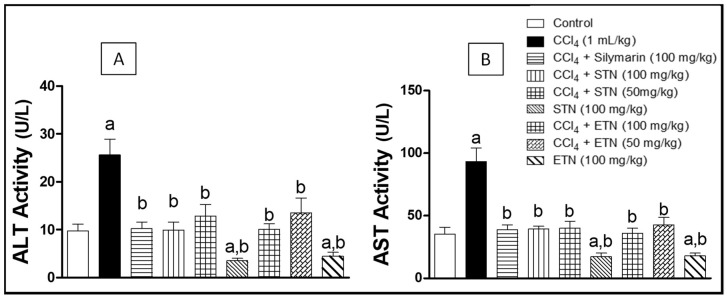
Effect of ETN and STN on ALT (Panel **A**) and AST (Panel **B**) serum activities in rats subjected to chronic CCl_4_ intoxication. * Data are the mean ± SD (*n* = 10). a or b Significantly different from control or CCl_4_ group respectively at *p* < 0.05 using ANOVA followed by Tukey-Kramer as a post-hoc test.

The exposure to CCl_4_ caused a significant 3-fold increase in the serum AST and ALT levels by when compared to the control group. Co-administration of silymarin with CCl_4_ significantly lowered the AST and ALT levels near to the normal levels. Moreover, the concurrent administration of ETN with CCl_4_ caused a dose related decrease in the levels of the AST and ALT by 61% and 60% respectively as compared to the CCl_4_ intoxicated group. Similarly, the simultaneous administration of the hydro alcoholic extract of STN with CCl_4_ caused a dose related lowering in the levels of the AST and ALT by 60% and 58% respectively in comparison with the CCl_4_- challenged group. Remarkably, the treatment of the rats with the ETN or STN extract alone caused a significant decline in the AST and ALT concentrations as compared to the control group (Figure 2).

### 2.5. Histopathological Examination with Hematoxylin and Eosin (H and E) Stain

**Figure 3:**
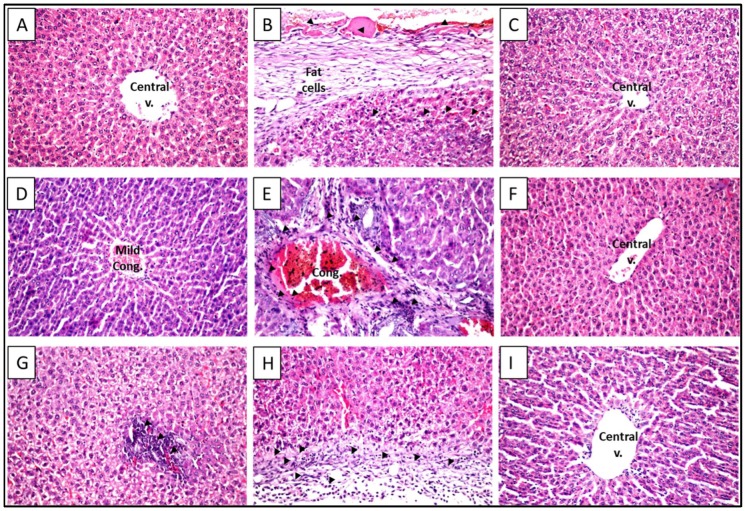
Representative photomicrographs of liver sections stained with (**H**&**E**) (100×). (**A**) Section taken from a control rat liver showing normal central vein and hepatic architecture; (**B**) Section taken from rat liver exposed to CCl_4_ showing thickening and fibrosis with fat cells deposition in the hepatic capsule associated with extended fibrosis to the hepatic parenchyma between the degenerated hepatocytes (arrowhead); (**C**) Section taken from rat liver exposed to CCl_4_ and treated with silymarin (100 mg/kg) showing restoration of normal histological structure; (**D**) Section taken from rat liver exposed to CCl_4_ and treated with ETN (100 mg/Kg) showing mild congestion in the central vein; (**E**) Section taken from rat liver exposed to CCl_4_ and treated with ETN (50 mg/Kg) showing portal vein congestion with inflammatory cells infiltration in the portal area; (**F**) Section taken from rat liver exposed only to ETN (100 mg/Kg) showing no histopathological alterations; (**G**) Section taken from rat liver exposed to CCl_4_ and treated with STN (100 mg/Kg) showing focal inflammatory cells infiltration (arrowheads); (**H**) Section taken from rat liver exposed to CCl_4_ and treated with STN (50 mg/Kg) showing diffuse inflammatory cells infiltration (arrowheads), with hepatocytes degeneration. (**I**) Section taken from rat liver exposed only to STN (100 mg/Kg) showing normal liver architecture.

Liver sections taken from the control group stained with H&E displayed a normal histological structure of the central vein and surrounding hepatocytes with no histopathological alterations (Figure 3A). Treatment with ETN or STN only didn’t exhibit any change in the normal liver architecture (Figure 3F,I). Exposure to CCl_4_ triggered a thickening and fibrosis with fat cells deposition in the hepatic capsule associated with extended fibrosis to the hepatic parenchyma between the degenerated hepatocytes, in addition to the portal area that showed also fibrosis which was extended to the parenchyma between the degenerated hepatocytes with inflammatory cells infiltration and congestion in the portal vein (Figure 3B). The rats concurrently treated with silymarin showed restoration of normal histological structure (Figure 3C). Animals co-treated with ETN (100 mg/kg) presented a mild congestion in the central vein (Figure 3D), while ETN lower dose showed portal vein congestion with inflammatory cells infiltration in the portal area (Figure 3E). On the other hand, groups co-administered STN (100 mg/kg) showed only focal infiltration with inflammatory cells (Figure 3G), while STN co-treatment (50 mg/kg) revealed diffuse cell infiltration with hepatocellular degeneration (Figure 3H). 

### 2.6. Oxidative Stress Parameters

**Figure 4:**
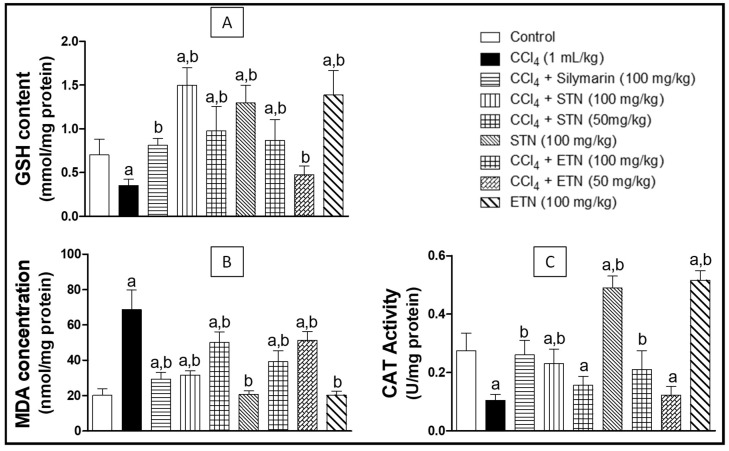
Effect of ETN and STN on hepatic GSH content (Panel **A**), lipid peroxidation as MDA concentration (Panel **B**); Catalase enzymatic activity (Panel **C**) in rats subjected to chronic CCl_4_ intoxication. * Data are the mean ± SD (*n* = 10). a or b: Significantly different from control or CCl_4_ group respectively at *p* < 0.05 using ANOVA followed by Tukey-Kramer as a post-hoc test.

As shown in Figure 4A the CCl_4_ intoxication significantly reduced the GSH level by 50% as compared to the control group. Concomitant treatment with silymarin and CCl_4_ offered a significant protection against CCl_4_ intoxication by significantly increasing the GSH level reaching 230% as compared to the CCl_4_ groups. Furthermore, animals treated with the ETN or STN extract along with CCl_4_ showed an increase in the level of GSH by 147% and 328% respectively when compared to group exposed to CCl_4_ in a dose related manner_._ Remarkably, the administration of the ETN or STN alone revealed a significant increment in the level of GSH reaching 214% and 185% in comparison to the control group.

Lipid peroxidation measured as malondialdehyde (MDA) concentration showed a significant 3-fold increase as a result of CCl_4_ administration, as compared to the control group. The administration of silymarin in tandem with CCl_4_ exhibited a significant inhibition in the rise of MDA level and kept it within the normal values when compared to the control. Moreover, animals treated with the ETN or STN extract in addition to CCl_4_ showed a dose related reduction in the level of MDA by 42% and 54% respectively when compared to CCl_4_ intoxicated group_._ On the other hand, there was no significant change in the lipid peroxidation when the animals were administered the ETN or STN extract alone in comparison with the control group (Figure 4B).

Intoxication with CCl_4_ brought an evident reduction in the catalase activity by 62% as compared to the control group. Treatment of animals with silymarin concomitantly with CCl_4_ triggered significant increased the catalase activity level to reach 248% as compared to the CCl_4_-exposed group. Moreover, animals treated by ETN or STN extract along with CCl_4_ exhibited a dose related increase in the activity of catalase reaching 200% and 224% respectively when compared to group exposed to CCl_4_. Interestingly The administration of the ETN or STN alone revealed a significant raise in the catalase activity by 88% and 81% after comparison with the control group (Figure 4C).

### 2.7. Liver Fibrosis Markers

**Figure 5:**
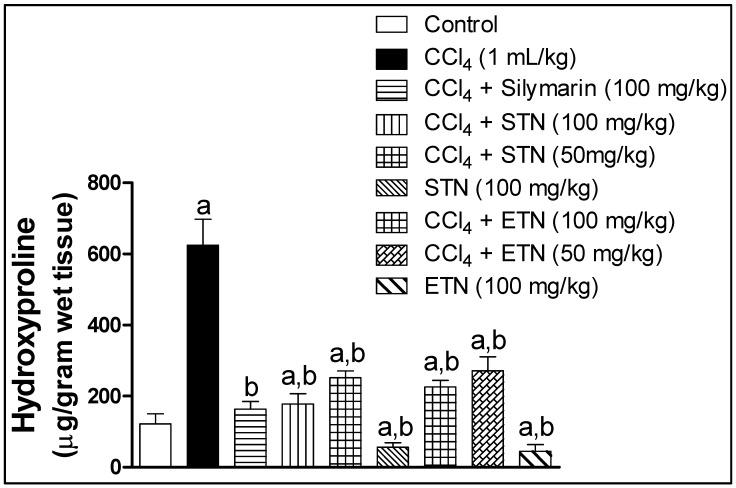
Effect of ETN and STN on liver hydroxyproline content rats subjected to chronic CCl_4_ intoxication. * Data are the mean ± SD (*n* = 6). a or b: Significantly different from control or CCl_4_ group respectively at *p* < 0.05 using ANOVA followed by Tukey-Kramer as a post-hoc test.

Liver fibrosis was assessed biochemically by determining collagen accumulation indices in terms of its main component, hydroxyproline. Measurement of hydroxyproline assured the histological observation of enhanced liver fibrosis by CCl_4_. As the liver hydroxyproline content in the CCl_4_ challenged group was 513% as compared to the control group. The animal treatment with silymarin together with CCl_4_ significantly lowered the hydroxyproline content in liver by 73% with respect to the CCl_4_ exposed group. Moreover, animals treated with the ETN or STN extract in addition to CCl_4_ showed a dose related significant reduction in the level of liver hydroxyproline by 64% and 71% respectively when compared to CCl_4_ intoxicated group_._ On the other hand, there was no significant alteration in the hydroxyproline liver content when the animals were administered the ETN or STN extract alone in comparison with the control group (Figure 5).

**Figure 6:**
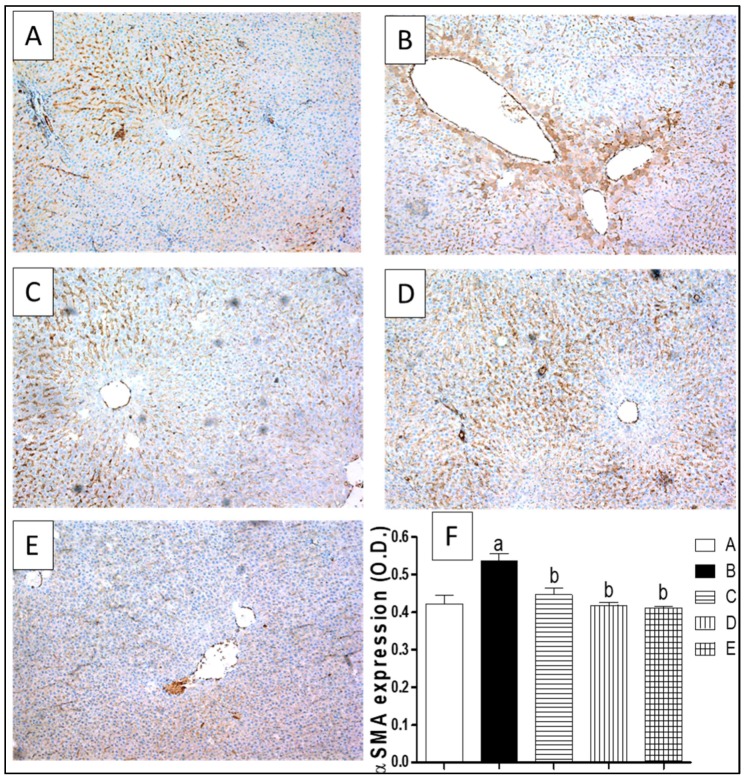
Expression of alpha smooth muscle actin (α-SMA) by immunohistochemical staining (×100). (**A**) Photomicrograph of liver section of control rats showing minimal immunostaining for *α*-SMA; (**B**) Photomicrograph of liver section of CCl_4_ intoxicated rats showing extensive *α*-SMA expression of as shown by the intense brown staining; (**C**) Photomicrograph of liver section of (CCl_4_/Silymarin) treated rats showing limited *α*-SMA expression; (**D**) Photomicrograph of liver section of rats concurrently treated with CCl_4_ (1 mL/kg) twice a week and ETN (100 mg/kg) three times per week, showing limited *α*-SMA expression; (**E**) Photomicrograph of liver section of rats simultaneously treated with CCl_4_ (1 mL/kg) twice a week and STN (100 mg/Kg) three times per week, showing minimal *α*-SMA expression; (**F**) A graphical representation of the *α*-SMA expression as optical density (O.D) for the liver sections from different groups, where a or b express the significant difference from control or CCl_4_ group respectively at *p* < 0.05 using ANOVA followed by Tukey-Kramer as a post-hoc test.

The immunohistochemical examination of α-SMA expression revealed minimal staining in the blood vessels of the control group (Figure 6A). A marked expression was observed periportally and perisinusoidally in the CCl_4_ exposed group as shown by the intense brown staining and optical density (O.D) of 127% with respect to the control group (Figure 6B). Animals co-treated with silymarin, ETN or STN extract markedly attenuated this elevated expression (Figure 6C–E) which was assured by lowering the O.D to 83%, 80% and 77% as compared to the CCl_4_ exposed group.

### 2.8. Inflammatory Markers

**Figure 7:**
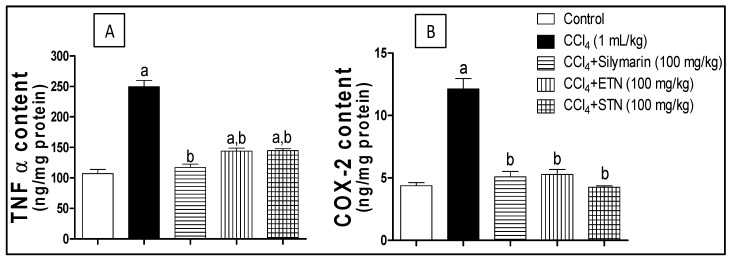
Effect of ETN and STN aqueous alcohol extracts on hepatic TNF-*α* (Panel **A**) and COX-2 content (Panel **B**) in rats subjected to chronic CCl_4_ intoxication. * Data are the mean ± SD (*n* = 6). a or b: Significantly different from control or CCl_4_ group respectively at *p* < 0.05 using ANOVA followed by Tukey-Kramer as a post-hoc test.

The measurement of the TNF-α and COX-2 content was carried out using ELISA technique. Exposure to CCl_4_ caused a significant rise in the TNF-α value by 133% with respect to the control group. The concurrent treatment with silymarin, ETN or STN suppressed the rise in the TNF-α by 53%, 42.5% and 42% respectively, in comparison with the CCl_4_ challenged group (Figure 7A).

The COX-2 levels showed a similar pattern to that of TNF-α by a 3-fold increase in the COX-2 concentration upon exposure to CCl_4_.The effect of the intoxication was ameliorated by co- administration of silymarin, ETN or STN that reduced the COX-2 level to 41%, 43% and 35% respectively, as compared to the group intoxicated with CCl_4_ (Figure 7B).

**Figure 8:**
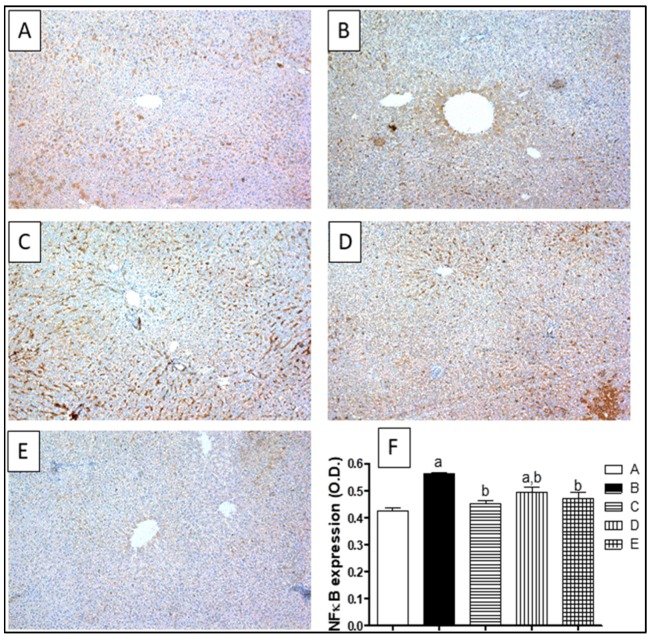
Expression of nuclear factor kappa B (NF-κB) by immunohistochemical staining (100×). (**A**) Photomicrograph of liver section of control rats showing minimal immunostaining for NF-κB; (**B**) Photomicrograph of liver section of CCl_4_ intoxicated rats showing increased NF-κB expression of as shown by the intense brown staining; (**C**) Photomicrograph of liver section of (CCl_4_/silymarin) treated rats showing limited NF-κB expression; (**D**) Photomicrograph of liver section of rats concurrently treated with CCl_4_ (1 mL/Kg) twice a week and ETN (100 mg/Kg) three times per week, showing limited NF-κB expression; (**E**) Photomicrograph of liver section of rats simultaneously treated with CCl_4_ (1 mL/Kg) twice a week and STN (100 mg/Kg) three times per week, showing decreased NF-κB expression; (**F**) A graphical representation of the NF-κB expression as optical density (O.D) for the liver sections from different groups, where a or b express the significant difference from control or CCl_4_ group respectively at *p* < 0.05 using ONE-WAY ANOVA followed by Tukey-Kramer as a post-hoc test.

NF-κB was assessed immunohistochemically by detecting the activated subunit p65 in liver tissues. Control rats showed a minimal immunostaining for NF-κB (Figure 8A). CCl_4_ brought an increase in the p65 content in the liver tissues, which was manifested by the intense brown staining that was proven by the significant increase in OD by 33% (Figure 8B). However, silymarin significantly lowered the expression of NF-κB as well as the OD by 19% with respect to the CCl_4_-challenged group. In addition, co-treatment of rats with ETN or STN significantly reduced the expression of NF-κB, which was assured by the reduction in the OD by 12% and 16%, respectively, when compared to CCl_4_-intoxicated group (Figure 8D,E).

## 3. Discussion

The aim of current study was to explore the metabolite profile of the alcohol soluble fraction of *T. nilotica* aqueous extracts (ETN and STN), assess in vivo antifibrotic activity and the possible underlying mechanism. HPLC/HRESI/MS/MS profiling of ETN and STN highlighted the presence of various polyphenol derivatives, which played an important role in the antioxidant and antifibrotic activities. Interestingly, sulphated polyphenols represented the main components of STN in comparison to ETN. This finding is due to the fact that, same species growing in different geographical locations is subjected to variations due to their occurrence in contrasting ecological and environmental conditions [22]. Despite the fact that both ETN and STN exhibited potential antifibrotic activity; the results displayed a significant GSH elevation in animal groups treated with STN when compared to that treated with ETN or even the control rats. This could be referred to the high content of sulphated polyphenols present in STN, which assure the fact reported earlier that the intake of sulphated polyphenols significantly influence GSH synthesis [23]

Polyphenols are well-known for their antioxidant and anti-inflammatory properties on both in vitro and in vivo experimental models [24]. Accumulating evidence shows that inflammation—in addition to increased oxidative stress—plays an important role in the development of different liver diseases including fibrosis [25]. According to the current and former study results, *T. nilotica* is reported as a rich source of various polyphenol compounds [26]. In the present study, the ORAC assay results stand in line with the calculated total phenolic content, showing that the radical scavenging capacity of ETN and STN fractions could be a result of the high phenolic content of both extracts. 

Concerning the antifibrotic activity, ETN and STN were investigated against CCl_4_-induced fibrosis in rats. In this regard, CCl_4_ is considered as one of the most regularly used hepatotoxins in the experimentally-induced liver diseases in animals [27]. Its hepatotoxic effect is initiated by cytochrome P450 2E1 that converts CCl_4_ to the highly reactive trichloromethyl radical (CCl_3_**^·^**) which is afterward transformed into more destructive trichloromethyl peroxy radical (CCl_3_OO^·^) in the presence of oxygen [28]. The generated free radicals cause injury of hepatocellular membrane via depleted GSH content, increased lipid peroxidation, and release of inflammatory mediators from activated inflammatory cells that in turn potentiate CCl_4_-induced hepatic injury [29]. 

In the present study, CCl_4_ caused a significant elevation in serum ALT and AST, indicating hepatocellular inflammation and necrosis [30]. Co-treatment of animals with ETN or STN significantly alleviated such progressive hepatic changes. Silymarin was used as a standard antifibrotic agent due to its well-known hepatoprotective ability [31]. These effects were further proven by histopathological examination of the CCl_4_ animals co-treated with the extract or silymarin, which presented minimal hepatic tissues disfiguring as compared to the CCl_4_-exposed rats. 

Lipid peroxidation (measured as MDA) is recognized as one of the principal steps of CCl_4_-induced liver injury, as a result, the antioxidant activity is a very important property in a hepatoprotective agents. In fact, MDA has been used for a long time as a biomarker of oxidative stress [32,33]. The increase of MDA reflects enhanced lipid peroxidation and tissue injury. Catalase (a tetrameric heme protein), is one of the major intracellular antioxidant enzymes. Its function is to protect cells from the accumulation of H_2_O_2_ by catalyzing its decomposition into water and oxygen [34]. The reduced catalase (CAT) activity is a proof for compromised enzymatic protection against tissue damage caused by elevated oxidative stress. In contrast, GSH represents the non-enzymatic part of the host antioxidant defense mechanism. It can effectively scavenge free radicals, while being oxidized by glutathione peroxidase into glutathione disulfide which can be reduced back to GSH by glutathione reductase with the consumption of NADPH [35]. GSH can also react with various electrophiles, physiological metabolites and xenobiotics to form mercapturates, which are catalyzed by other antioxidant enzymes. 

In this study, there was a marked increase in MDA with concomitant decrease in GSH and CAT activity after chronic CCl_4_ challenge. However, ETN and STN extracts and standard silymarin kept the MDA levels close to or within the normal values. In another context, ETN, STN and silymarin triggered a significant increase in the CAT activity making it closer to the normal values. As for the GSH content, the silymarin and ETN prevented its consumption, while STN significantly raised the GSH level even above control which could count for the STN efficient antioxidant activity. The observed results are in agreement with earlier studies that showed that several antioxidants possess potential protective effects against CCl_4_ induced liver fibrosis [36,37].

Interestingly, NF-κB is a transcriptional regulator of genes involved in immunity, inflammatory response, cell fate, and function [38]. Kupffer cells display powerful NF-κB activation in response to liver injury by CCl_4_ and the oxidative damage. This results in production and secretion of proinflammatory cytokines such as TNF-α which is strongly implicated as a fibrosis promoter [39]. Accordingly, the persistent elevation in the levels of NF-κB promote the secretion of inflammatory and chemotactic factors in hepatocytes and thereby worsen hepatic inflammation and fibrosis [40]. In this context, several studies showed that downregulation of NF-κB expression and subsequent inflammatory cascade is capable of alleviating CCl_4_-induced hepatic fibrogenesis [41,42]. Of the various kinds of inflammatory mediators TNF-α plays an important role in the pathogenesis of liver fibrosis through activation of Kupffer cells. TNF-α stimulates the release of cytokines from the macrophages and induces phagocyte oxidative metabolism [43]. Thus, a vicious cycle is established in the hepatocytes: TNF-α promotes NF κB activation, and NF-κB leads to enhanced production of additional TNF-α. This cycle eventually alters the structure of the hepatocytes, and impairs their function, consequently, prolonged activation of NF-κB leads to perpetuated inflammatory responses [44]. Moreover, the upregulation of COX-2 has been demonstrated in human liver cirrhosis as a result of active inflammation [45,46]. In the current study, the administration of silymarin, ETN and STN concomitantly with CCl_4_ significantly reduced the expression of NF-κB and hence inhibited the downstream inflammatory cascade as evidenced by the significant inhibition of the augmented hepatic levels of TNF-α and COX-2 demonstrating the satisfactory anti-inflammatory properties of the extracts.

Hydroxyproline is one of the sensitive markers that significantly rises during fibrosis reflecting the increase in the synthesis of collagen [47]. In addition to collagen, expression of the microfilament protein “α-SMA” has been explored as a marker for activation hepatic stellate cells and hence hepatic fibrosis [48]. In this study, there was a significant increase in the hydroxyproline content associated with α-SMA overexpression due to CCl_4_ exposure. Co-administration of ETN, STN or silymarin offered a decrease in the hydroxyproline level along with reduction in the α-SMA expression. It is important to note that all of the effects caused by the co-treatment with ETN or STN were dose dependent. 

From the available results, it could be concluded that the ETN and STN presents a significant in vivo dose related antifibrotic activities, which in turn may be due to the polyphenols content. The underlying mechanism could be—at least partly—due to restraining of the oxidative stress through GSH replenishment along with inhibiting the inflammatory response. These findings stands in line with previous reports that confirms the antifibrotic activity of quercetin [49,50], kaempferol [51], gallic acid [52] or falvonoids [53]. In addition to the earlier report which refer the in vivo hepatoprotective properties of *T. nilotica* flowers to its phenolic constituents [8].

## 4. Materials and Methods

### 4.1. Materials

The aerial parts of *T. nilotica* were collected from the campus of the German University (Cairo, Egypt) and from Al-Taif Mountain (Saudi Arabia) in March, 2015. Both plants were collected at the flowering stage. The authenticity of the species was confirmed by Dr. Mohamed El Gebaly (Professor of Taxonomy at the National Research Center, Egypt). A voucher specimen was deposited at the Herbarium of Department of Pharmacognosy, Faculty of Pharmacy, Ain Shams University, Egypt. Sample was kept under voucher number PHG-P-TN194. Ellman’s reagent [5,5-dithio-bis(2-nitrobenzoic acid); DTNB], reduced glutathione (GSH), bovine serum albumin, chloramine-T, *p*-dimethylaminobenzaldehyde (PDMA), hydroxyproline, and thiobarbituric acid (TBA) were purchased from Sigma-Aldrich Chemical Co. (St Louis, MO, USA). Carbon tetrachloride (CCl_4_), *n*-butanol, dipotassium hydrogen phosphate (K_2_HPO_4_), potassium dihydrogen phosphate (KH_2_PO_4_) and trichloroacetic acid (TCA) were obtained from Al-Gomhoryyah Chemical Co. (Cairo, Egypt).

### 4.2. Animals

Animal experiments were conducted in accordance with the ethical guidelines of Ain Shams University (Cairo, Egypt). Male albino rats (100–150 g) were obtained from Nile Co. for Pharmaceutical and Chemical Industries (Cairo, Egypt). Rats were housed in an air-conditioned atmosphere, at a temperature of 25 °C with alternatively 12 h light and dark cycles. Animals were acclimatized for 2 weeks before performing the study. They were kept on a standard diet and water *ad libitum*. Standard diet pellets (El Nasr, Cairo, Egypt) contained not less than 20% protein, 5% fiber, 3.5% fat, 6.5% ash and a vitamin mixture. Animal care and experimental design were approved and conducted in accordance with the guidelines approved by the Research Ethics Committee, Faculty of Pharmacy, Ain Shams University, Egypt. (#00146/15).

### 4.3. Plant Extraction

*Tamarix nilotica* aerial parts (750 g) from Egypt and Saudi Arabia were exhaustively extracted with distilled water at 25 °C with stirring (2 L × 3), individually. Each extract was dried at 40 °C under vacuum followed by alcohol extraction. The alcohol soluble fractions of aqueous extracts were dried under vacuum to yield 40.36 g and 44.71 g or residue, respectively. The alcohol soluble fraction of aqueous extract of Saudi *T. nilotica* (STN) and Egyptian *T. nilotica* (ETN) were subjected to further analysis by HPLC/HRESI/MS/MS.

### 4.4. Phenolic Content

The total phenolic content of the extract was assessed through Folin–Ciocalteu method [54]. A volume of 200 μL of crude extract (1 mg/mL) were diluted with distilled water to 3 mL then mixed thoroughly with 0.5 mL of Folin–Ciocalteu reagent for 3 min, followed by the addition of 2 mL of 20% (*w*/*v*) anhydrous sodium carbonate. The mixture was allowed to stand for 60 min in the dark, and absorbance was measured at 765 nm. The calculation of the total phenolic content was done using gallic acid as a standard via calibration curve with a straight line equation (y = 0.0012x + 0.5958) and regression correlation (R^2^ = 0.9994). Results (Table 3) are presented in equivalent milligrams of gallic acid per 1.0 g of dried extract. All assays were carried out in triplicates [55].

### 4.5. LC–HRESI-MS–MS Analysis

The chromatographic analysis was performed on an Agilent 1200 series HPLC instrument (Agilent Technologies, Santa Clara, CA, USA), the column was a Gemini 3 µm C18 110 Å (Phenomenex, Torrance, CA, USA), with dimensions 100 × 1 mm i.d., protected with a RP C18 100 Å guard column with dimensions 5 mm × 300 µm i.d., 5 µm. The mobile phase components were 2% acetic acid (A) and 90% MeOH, 2% acetic acid (B) at a flow rate of 50 μL/min. The mobile phase gradient was: 0–60 min, 5% B; 60–70 min, 50% B; 70–80 min, 90% B; 80–90 min, 5% B. The samples were dissolved in 5% MeOH and 2% acetic acid with a concentration of 1 mg/mL then filtered using a syringe filter with a pore size 0.2 µm. The sample injection volume was 10 μL. A Fourier transform ion cyclotron resonance (FTICR) mass analyzer was used equipped with an electrospray ionization (ESI) system and controlled by Xcalibur^®^ software. Detection was performed in the negative and positive ion modes applying a capillary voltage of 36 V and a temperature of 275 °C. The API source voltage was adjusted to 5 kV, and the desolvation temperature to 275 °C. Nitrogen was used as a nebulizing gas with a flow adjusted to 15 L/min. The analytical run time was 89 min and the full mass scan covered the mass range from 150 to 2000 *m*/*z* with resolution of 100,000 [55]. Quantitation by LC-MS-MS; the absolute area and area percent of each peak was calculated by Xcalibur^®^ software with ICIS peak algorithm then exported to Microsoft Excel^®^ software for preparation of graphs and further data analysis.

### 4.6. Oxygen Radical Absorbance Capacity (ORAC Assay)

Experiments were performed as previously described in black 96-well plates [56]. In each well of a 96-well plate, 150 µL fluorescein (final concentration: 2.5 nM), 25µL Trolox (final concentrations: 0.78–25 µM) or 25 µL tested samples were pipetted in quadruplicate. Plate was allowed to equilibrate at 37 °C for 30 min. After this time, fluorescence measurements (Ex. 485 nm, Em. 520 nm) were taken every 90 s; first to determine the background signal. After three cycles 25 µL AAPH (final concentration: 60 mM) were added manually in each well with a multi-channel-pipette. Measurements were taken as quickly as possible since the ROS generator displays immediate activity after addition. Fluorescence measurements were continued for 90 min. Half-life time of fluorescein was determined using the MS Excel software.

### 4.7. In-vivo Experimental Design

Animals were classified randomly into nine groups (ten animals per group) and treated for six weeks as follows; Group 1: Rats were given corn oil three times per week and considered as control animals; Group 2: Rats were given CCl_4_ (1 mL/kg, 1:1 mixture with corn oil, i.p.), twice weekly to induce liver fibrosis [57]; Group 3: Rats were given CCl_4_ (1 mL/kg, 1:1 mixture with corn oil, i.p.) twice weekly and silymarin oral suspension (100 mg/kg, suspended in distilled water), three times per week at alternating days with CCl_4_ and considered as a positive control; Group 4: Rats were given CCl_4_ (1 mL/kg, 1:1 mixture with corn oil, i.p.) twice weekly and ETN extract (100 mg/kg, orally, dissolved in distilled water), three times per week at alternating days with CCl_4_; Group 5: Rats were given CCl_4_ (1 mL/kg, 1:1 mixture with corn oil, i.p.) twice weekly and ETN extract (50 mg/kg, orally, dissolved in distilled water), three times per week at alternating days with CCl_4_; Group 6: Rats were given ETN extract alone (100 mg/kg, orally, dissolved in distilled water) three times per week; Group 7: Rats were given CCl_4_ (1 mL/kg, 1:1 mixture with corn oil, i.p.) twice weekly and STN extract (100 mg/kg, orally, dissolved in distilled water), three times per week at alternating days with CCl_4_; Group 8: Rats were given CCl_4_ (1 mL/kg, 1:1 mixture with corn oil, i.p.) twice weekly and STN extract (50 mg/kg, orally, dissolved in distilled water), three times per week at alternating days with CCl_4_; Group 9: Rats were given STN alone (100 mg/kg, orally, dissolved in distilled water) three times per week.

At the end of the sixth week, the rats were anaesthetized and blood samples were collected from the retro-orbital plexus and allowed to clot. Serum was separated by centrifugation at 1000 rpm for 10 min and used for the assessment of liver functions. Then, rats were sacrificed and liver tissues were dissected, weighed and washed with ice-cold saline. Then livers were then homogenized in ice-cold saline using a homogenizer to obtain 20% homogenate. Aliquots of the liver homogenate were stored at −80 °C prior to biochemical analysis. In addition, specimens from the three major lobes of each liver were fixed in 10% formalin saline for histopathological and immunohistochemical investigations.

### 4.8. Assessment of Hepatotoxicity Indices

Serum aspartate aminotransferase (AST) and alanine aminotransferase (ALT) were determined according to the procedure previously reported [57].

### 4.9. Assessment of Oxidative Stress Markers

For evaluating GSH reserves, 0.5 mL of the homogenate was added to a tube with 0.5 mL of 10% TCA. For 15 min, the tubes were shaken moderately and intermittently, then a centrifugation at 3000 rpm for 10 min was done. An aliquot of the formed supernatant (0.2 mL) was added to a tube containing 0.1 mL Ellman’s reagent and 1.7 mL phosphate buffer, then the absorbance was read at 412 nm within 5 min [58]. The results were expressed as mmol of GSH/mg protein. Lipid peroxidation was measured by calculating the level of thiobarbituric acid reactive substances (TBARS) measured as malondialdehyde (MDA), as per the method of [59]. The reaction was prepared by addition of 0.5 mL of the homogenate to 1.0 mL 0.6% TBA and 2.5 mL of 20% TCA, and then the mixture was heated in a boiling water bath for 20 min followed by cooling and addition of 4 mL *n*-butanol along with shaking. Separation of the alcohol layer was done by 10 min centrifugation at 2000 rpm for and absorbance was measured at 535 nm. The results were expressed as nmole of MDA/mg protein using 1,1,3,3- tetraethoxypropane used as standard. In addition, CAT activity was assessed using commercially available biochemical kit (Biodiagnostics, Cairo, Egypt).

### 4.10. Assessment of Liver Fibrosis

Liver fibrosis was evaluated using two different markers: hydroxyproline and α-SMA. The first one was determined according to previously described method [60]. The procedure utilized a volume of 0.5 mL of 20% liver homogenate which was kept in 1 mL of 6 M HCl for 8 h at 120 °C. A portion of the digested homogenate (25 μL) is added to 25 μL citrate-acetate buffer then 500 μL of chloramine T solution was added and finally the mixture is kept for 20 min at room temperature. Then, 500 μL Ehrlich’s solution was added and the mixture is incubated at 65 °C for 15 min. After cooling for 10 min, the color developed was spectrophotometrically measured at 550 nm. The results were presented as μg/g of wet tissue. In addition, α-SMA expression in formalin-fixed paraffin-embedded rat liver was assessed immunohistochemically using monoclonal antibody (MA1-744, ThermoFisher, Loughborough, UK).

### 4.11. Assessment of Inflammatory Markers

The TNF-*α* and COX-2 levels in liver homogenate was performed using commercial ELISA kit obtained from Sigma Aldrich Chemical Co. according to the manufacturer's instructions. The quantities of TNF-*α* and COX-2 were expressed as ng/mg protein. The protein was calculated using bovine serum albumin as standard [61]. Furthermore, NF-κBp65 subunit expression was assessed immunohistochemically in formalin-fixed paraffin-embedded rat liver using polyclonal antibody (PA5-16545, ThermoFisher).

### 4.12. Statistical Analysis

Data are presented as mean ± SEM, multiple group comparisons were carried out using one-way analysis of variance (ANOVA) followed by the Tukey-Kramer test for post-hoc analysis. Probability values of *p* < 0.05 were considered statistically significant. All statistical analyses were performed using GraphPad InStat software, version 3.05 (GraphPad Software, Inc., La Jolla, CA, USA). Graphs were sketched using GraphPad Prism software, version 5.00 

## Figures and Tables

**Table 1:** Characterization of metabolites from ETN by HPLC-MS/MS in negative ion mode.

#	Rt (Min)	[M − H]^−^	Error (ppm)	MS/MS Fragment	Formula	Identity	References
1	3.9	169.0220	−2.9	125.1086	C_7_H_6_O_5_	Gallic acid	[12,13]
2	4.12	301.0421	1.6	273.0435, 179.0096, 150.9980	C_15_H_10_O_7_	Quercetin	[15,18]
3	6.59	483.0859	−1.2	331.1573, 313.1711, 169.1114	C_20_H_20_O_14_	di-Galloyl-glucose (nilocitin)	[14]
4	14.72	285.0469	2.8	239.0530, 143.1847	C_15_H_10_O_6_	Kaempferol	[15,18]
5	28.09	197.0531	−1.5	183.2035, 182.1017, 168.1108, 167.1539	C_9_H_10_O_5_	Methyl gallate methyl ether	[14]
6	30.61	259.0356	−0.8	229.0872, 179.0628,	C_10_H_12_O_6_S	Coniferyl alcohol sulphate	[11]
7	36.82	193.0574	2.6	178.1750, 149.1777, 134.0983	C_10_H_10_O_4_	Ferulic acid isomer	[17,19]
8	42.4	477.1101	2.1	315.0990, 300.1015	C_22_H_22_O_12_	Methylquercetin hexoside (tamarixetin-3-*O*-hexoside)	[16]
9	43.93	273.0145	0.7	229.0810, 193.1538, 178.0940	C_10_H_10_O_7_S	Ferulic acid sulphate derivative	[10]
10	53.62	314.1315	−0.32	177.0162, 164.1282, 145.1110	C_18_H_19_NO_4_	*n*-Feruloyltyramine *	[20,21]
11	57.08	287.0299	1.4	272.1937, 207.1997, 192.1747	C_11_H_12_O_7_S	Methyl ferulate sulphate	[9]
12	58.3	461.0806	−1.7	285.1083, 257.3536	C_21_H_18_O_12_	Kaempferol glucuronide	[15]
13	67.53	315.0587	−1.3	300.1302, 193.1954	C_16_H_12_O_7_	Methylquercetin (tamarixetin)	[9,16,17]
14	71.19	299.0624	3	284.1601, 271.2777	C_16_H_12_O_6_	Methylkaempferol (kaempferide)	[9]
15	74.7	395.0154	−0.8	315.1095, 300.2426, 217.0871	C_16_H_12_O_10_S	Methylquercetin-sulphate (tamarixetin sulphate)	[11,17]
16	75.12	379.0195	1.8	299.1220, 284.2777	C_16_H_12_O_9_S	Kaempferol methyl ether sulphate	[11]
17	75.89	393.0366	−2	313.1219, 298.0152, 283.8303	C_17_H_14_O_9_S	Kaempferol dimethyl ether sulphate	[15]

* Detected only through inspection of the positive ion mode. ETN is the alcohol soluble fraction of aqueous extract of *T. nilotica* from Egypt.

**Table 2:** Characterization of metabolites from STN by HPLC-MS/MS in negative ion mode.

#	Rt (Min)	[M − H]^−^	Error (ppm)	MS/MS Fragment	Formula	Identity	References
1	3.97	169.0213	1.18	125.1079	C_7_H_6_O_5_	Gallic acid	[12,13]
2	4.17	301.0425	0.33	273.0441, 179.0084, 150.9916	C_15_H_10_O_7_	Quercetin	[15,18]
3	6.47	483.0851	0.41	331.1561, 313.1709, 169.1116	C_20_H_20_O_14_	di-Galloylglucose (nilocitin)	[14]
4	14.79	285.0483	−2.10	239.0536, 143.1841	C_15_H_10_O_6_	Kaempferol	[15,18]
5	28.13	197.0533	−2.52	183.2027, 182.1022, 168.1119, 167.1531	C_9_H_10_O_5_	Methyl gallate methyl ether	[14]
6	30.45	259.0351	1.15	229.0876, 179.0632	C_10_H_12_O_6_S	Coniferyl alcohol sulphate	[11]
7	43.98	273.0151	−1.46	229.0806, 193.1527, 178.0947	C_10_H_10_O_7_S	Ferulic acid sulphate derivative	[10]
8	53.72	314.1319	−1.60	177.0161, 164.12874, 145.1103	C_18_H_19_NO_4_	*n*-Feruloyltyramine *	[20,21]
9	56.93	287.0301	0.69	272.1925, 207.2002, 192.1733	C_11_H_12_O_7_S	Methyl ferulate sulphate	[9]
10	58.20	461.0795	0.65	285.1096, 257.3548	C_21_H_18_O_12_	Kaempferol glucruonide	[15]
11	67.77	315.0589	−1.90	300.1311, 193.1967	C_16_H_12_O_7_	Methyl-quercetin (Tamarixetin)	[9,16,17]
12	71.17	299.0629	1.33	284.1607, 271.2769	C_16_H_12_O_6_	Methyl-kaempferol (Kaempferide)	[9]
13	74.65	395.0159	−2.02	315.1085, 300.2417, 217.0865	C_16_H_12_O_10_S	Methyl-quercetin-sulphate (Tamarixetin-sulphate)	[11,17]
14	75.14	379.0191	2.89	299.1221, 284.2782	C_16_H_12_O_9_S	Kaempferol-methyl ether-sulphate	[11]
15	75.60	393.0368	−2.54	313.1212, 298.0147, 283.8312	C_17_H_14_O_9_S	Kaempferol-dimethyl ether-sulphate	[15]

* Detected only through inspection of the positive ion mode. STN is the alcohol soluble fraction of aqueous extract of *T. nilotica* from Saudi Arabia.

**Table 3:** Total phenolic content of ETN and STN.

	Total Phenolic Content (mg GA/gm Dry Extract)
ETN	95.1
STN	111.8
